# Medical Letter-Bag

**Published:** 1919-05

**Authors:** 


### THE MEDICAL LETTER-BAG

A Department for Short Correspondence from Physicians
"Piece out our imperfections with your thoughts."--Shakespeare

### Practical Applications of Ethical Economics.

Texas Medical Journal:

Prior to this war various evolutionary forces, without intelligent aid or organized assistance on the part of the medical profession, gradually compelled and are still compelling the followers of medicine into:

1. Accepting the specialist.
2. Demanding hospital facilities.
3. Associating and segregating into more or less organized groups.
4. Establishing private clinics, as the Mayo Clinic and similar institutions.
5. Establishing, as just instituted by Columbia University of New York, a still more advanced form of scientific medical organization, a clinical laboratory.

The same evolutionary forces have caused the laity:

1. To form mutual benefit organizatons for the sick.
2. To demand contract practice.
3. To form hospital associations.
4. To demand state aid.
5. To demand free clinics.

In every one of these vitally important politico-economic movements, and in view of the fact that millions of men will return after the war and demand for themselves and for their families the same scientific treatment they have experienced under military organization, medicine as a profession has failed to recognize the same exciting cause in each instance—an economic demand that the theoretical standard of efficiency "Medical Ethics" must be replaced by a more practical standard, "Ethical Economics." This standard demands the application of scientific methods through economic organization to every-day life, so that efficient medical and surgical treatment will come within the reach not of the few who can receive hospital treatment in standard institutions but of every human being.

Confronted by the above politico-economic facts, a very pertinent question presents itself to the medical profession at large:

What is medical organization—medical education—doing to solve these problems, at a time when an imminent reconstruction period confronts every form of organized society including the profession of medicine?

Based on observation and experience of 20 years the writer claims that virtually nothing practical has been systematically undertaken.

Nowhere is there evidence that medical organization—medical education—has ever recognized three basic psychologic factors that govern all intelligent human acts:

1. One hundred per cent. of the representatives of medicine, physicians, are human beings, and the minds of the highest and lowest are compounded of the same elements, held subject to the same laws of action; and the knowledge that any one of them possesses comes—as it does to every other human being—through the ordinary channels of the senses.

2. In the search for knowledge in every branch of human society, including medicine, science has produced innumerable mechanical aids to increase the efficiency of the senses of man. Therefore logically, all things being equal, the mind of man gathers knowledge in proportion (a) to the number of mechanical aids employed to increase the efficiency of the senses; (b) the accuracy with which these aids are employed.

3. As a rule normal human emotions govern every human being, including the physician. Therefore, if the recompense for labor does not enable the physician to carry overhead expenses; does not give him time and funds for improvement, study, travel and necessary recreation; does not produce profit that is protection for his family and for himself in sickness and old age; he can neither give efficient scientific service nor continue to progress. If adequately recompensed he can give scientific service far more readily and is more likely to progress.

Yet in the face of these obvious evolutionary politico-economic movements and the basic psychological facts that govern intelligent human action, medical education is still demanding for every individual admitted to the study of any branch of the science and art of medicine a high standard of preparatory education, in substance a B.A. degree from a recognized educational institution.

This standard, combined with the principles of education that are employed in every medical college after admission to study is such that it can be justly claimed that the educational methods pursued tend to make the graduate physician in this work-a-day world pursue the practice of medicine as a pure science, that can isolate itself, that needs no association with the applied sciences, especially economics. For instance, medical education during all these years has apparently never conceived of the practical necessity of recognizing the psychological fact No. 1 as a pre-educational factor of utmost importance.

The United States Government, on the other hand, by the present war has been unceremoniously forced into recognizing its educational value—as evidenced by the first standard of admission to the aviation service, where the highest possible human skill is required in order to successfully destroy life. In this initial examination the most accurate possible physical and mental tests are employed in order to ascertain not only the inherent character and personality of the candidate, but more especially the acuteness, stability and durability of *every one* of his senses.

In the profession of medicine, however, where there is a demand, if it were possible for even greater character and personality, acuteness, stability and durability of the senses—the object of the physician being to preserve life—no recognition is given to the fact that efficiency in applying abstract knowledge depends upon the efficiency, not of one but all of the special senses.

The student of medicine may be deficient in one or more of his special senses, have little tactile sensibility, a poor sense of smell or hearing, defective eyesight, little character and no personality adaptable to a physician. Yet no tests are made to ascertain or correct these defects, and the student is graduated and permitted without any organized supervision to try to preserve—where he would not on the same grounds be permitted to destroy—life.

As to the educational value of the psychological fact No. 2, there can be no question that the mechanical aids to scientific medicine (which include all laboratory methods, even history filing and compilation may be added) have become so numerous, have so developed in detail that to attain efficiency requires not general but definite technical knowledge.

There can be no question that medicine will become organized in the future, and when so organized it can be no exception to the general rule and must attain efficiency by having sub-division of labor—conference organization of labor and equipment.

Medical education as conducted today may be ethical but it is still decidedly theoretical. Medical schools virtually only graduate officers and then only colonels. No provision is made for officers of lesser rank, for the privates in the form of technicians. For privates, we as a profession, must take the unsuccessful physician, volunteer nurse, half-trained office girls, or any kind of unskilled help available, whom each physician must train for himself after his own sweet will in order to fill the rank of scientific medicine with privates. Yet economic organization is staring the profession in the face. With this army thus organized we guarantee to defend the public from disease —then wonder why our efforts as a profession are not appreciated

Even the colonel, who may later wish and be willing to work for a higher rank—for instance to become a specialist—there is no institution provided where through concentration of skilled leaders, equipment, technical assistance and economic organization he can learn his specialty from A to Z, and be instructed and equipped with a modified plan of economic organization, whereby he can do justice to the public and his profession by maintaining and delivering the high standard of goods which he advertises to sell in competition with the inferior grades of the cults by attaching to his name an "M. D."

As a profession, in most of our medical colleges, we unquestionably try to manufacture a high standard of goods, which goods must be sold in the open market to the public. We advertise to the public that the sign "M. D." signifies the highest standard. Yet as a profession have we adopted any organized means whereby we can demonstrate to the buyers, the laity, the value of standard "A" as compared with the imitation "B", and in so doing increase the demand for standard "A" goods, to the benefit of both producer and consumer? I think not.

The public through universal education is being taught to think, to reason, yet the medical profession today, like the cults, is asking the public to accept goods on faith without investigation; and we claim as a standard science based on reason, not wholly on faith.

If the profession of medicine will not undertake to solve these politico-economic problems for itself, it is true that evolutionary forces will solve them for us, but with brute force and a corresponding indiscriminate destruction—unless man employs the intelligence that nature has given him to anticipate evolutionary movements, through the use of intelligence scientifically applied but governed by the higher human emotions.

It is not within the limits of this letter even to outline the means to the end that experience suggests. But the old adage always proves true that where there's a will there's a way.

The object of this letter is to arouse, with your assistance, sufficient sentiment to instigate a systematic, organized movement to attain the end sought—the practical application of ethical economics; so that humanity may be efficiently served by the profession of medicine, and the profession win universal respect and attain efficiency through following out not only ethically but economically the dictates of the noblest of all the sciences.

Criticism of this suggestion is invited, and the writer would appreciate notice or information of any criticism or suggestion—direct or indirect—that may be offered.

Respectfully submitted,

G. Shearman Peterkin,

Cobb Building, Seattle, Wash.

---

### Spanish Influenza.

This disease has been so general all over the world that I feel sure there is not a physician engaged in general practice who has not had experience in treating it. As I said, this is a disease that has been prevalent all over the known world, confined generally to the human race, but I read recently that the monkeys in Africa were dying in large numbers from attacks of this disease. Does this substantiate Darwin's Theory?

As the disease has been so general I will not try to give its history, but confine myself to my personal experience with it, and will say now that I have never seen a case where the temperature remained above normal for more than three days, that serious complications did not develop. So general has this been the case with me, that I (with many others) call it "three-day fever." With me Pneumonia has been the most frequent and always in a very severe form, I have had as a complication or equally serious kidney trouble, also heart troubles of different kinds. While many of these pass away after the patients recover their strength, very many do not and are a cause of much anxiety to the physician and patients. Again, patients complain of soreness and stiffness in the joints. While there does not seem to be any redness or swelling of the parts, the patient complains of inability to use the limbs without pain, which at times is very acute. These conditions have usually passed away in two or three weeks after recovery. In *all* cases, that have come under my observation, there is a great prostration lasting from ten to thirty days. So general has this been with me that I insist on my patients remaining in bed at least five or six days after the fever has subsided and in the house not less than ten days.

In my experience this disease has a very peculiar effect on the female, a very large percentage of my female patients have the menstrual "flow" brought on by the disease regardless of the fact as to whether it is the regular period or not. I have had many patients tell me it had been only two weeks since they were "unwell" but their "sickness" was on them again. Very few of my patients who were ignorant passed through the disease without abortion. In proof of this I will say, as Local Registrar of Vital Statistics, I had more "stillborn" births reported to me from Oct. 1st, 1918, to March 1st, 1919, than in any twelve months during the several years I have acted in this capacity.

Have had two cases of acute mania following the disease, both negro women. One eluded her nurses and wandered around in the rain and mud in January (practically without clothes) for an hour before she was found and died in a few hours. The other case was a negro girl about 21 or 22 years of age. She had been treated by another physician for Flu. Two weeks after she had apparently recovered, she was brought in from the country and placed in my care. Found her with acute mania of a mild type; did not know any one, not even members of her own family; was much emaciated and so weak was almost helpless. With good nourishment and sedative tonics she recovered, after several weeks and is now well (April 25) physically and mentally and assisting in planting a crop.

Have had quite a number of cases at Otitis and Otoneura following the disease. At the March meeting of "The Clarksdale and Six Counties Medical Society," Dr. W. L. Howard of Memphis, Tenn., reported several cases of "Mastoid Inflamation as a result of Flu." While I had no cases of this kind in my practice, I have no doubt they came under Dr. Howard's observation, for I am of the opinion that Sherman's definition of the war will apply to Flu.

H. C. Buck, M. D.,
Friars Point, Miss.

---

**Answering Dr. DuBose's Question.**

---

Wells, Texas, May 7, 1919.

Texas Medical Journal:

In giving some light to Dr. DuBose's question in the April number of Texas Medical Journal concerning the value of pituritin in Post-Partum Hemorrhages. I shall say pituritin is recommended very highly in these conditions by Dr. Rushmore in the Bost. Med. & Surg. Jour. for Nov. 9, 1916, page 659, and Dr. E. Vogt, of Dresden.

Dr. W. A. Gardner, in the Jour. Kans. Med. Soc. July 1917, discourages the use of pituritin during the course of labor, but very favorably recommends its use in hemorrhages after the placenta has been passed.

I find that in some cases it gives better results than ergot. On one occasion where hemorrhage appeared in five or six days after confinement I had the most pleasing results with pituritin after ergot had failed.

And by the way, I have had success with it in treating incontinence of urine. Believing the majority of cases of incontinence of urine is caused by atony of the sphincter-muscle of the bladder, I used it for stimulation of involuntary muscular-fibers, and the results were good. After three or four subcutaneous injections, given once a week in doses of 2 to 1 mil., depending on the patient's age, incontinence of urine disappeared.

It is recommended by some in the treatment of Diabetes, Insipidus, Diuresis, Impotence in the male etc., but in these diseases I have never chanced to use it.

J. J. LOCKHART, M. D.

---

QUITE APPROPRIATE—Said She—Isn't that a duck of a bonnet Doctor Cubebs' wife has on?

Said He—Yes, and it's very appropriate.

Said She—How so?

Said He—Her husband is a "quack."

---

FATHER'S LIBERALITY:—Mrs. Youngwed (a doctor's daughter) —Did papa say he would do anything for you?

Youngwed—Yes, he said he would operate upon me at any time free of charge.—Pickings.

---

A WOODEN HEAD:—A Temple doctor gave a patient a bottle of liniment and told him to rub it thoroughly in the "lumbar regions." He applied it all on his head, but we guess he was right.

---

LOST OPPORTUNITY:—When our physician asked little Alice if she would come and live with him she said, "Doctor, you had me once; why didn't you keep me?"—Exchange.

